# Integrating Clinical Insights and Methodological Refinement: Addressing Key Limitations and Future Directions in Imaging Biomarkers

**DOI:** 10.1590/S1677-5538.IBJU.2025.0253

**Published:** 2025-05-30

**Authors:** Yuekun Fang, Shengyi Chen, Bin Cheng

**Affiliations:** 1 Wenzhou Hospital of Integrated Traditional Chinese and Western Medicine Department of Andrology Wenzhou China Department of Andrology, Wenzhou Hospital of Integrated Traditional Chinese and Western Medicine, Wenzhou, China; 2 Wenzhou Hospital of Integrated Traditional Chinese and Western Medicine Department of Urology Wenzhou China Department of Urology, Wenzhou Hospital of Integrated Traditional Chinese and Western Medicine, Wenzhou, China

To the editor,

We read with great interest the article by Vieira et al., titled "Comparison of Morphological and Functional MRI Assessments of Periprostatic Fat for Predicting Prostate Cancer Aggressiveness," published in the International Brazilian Journal of Urology (1). The authors present valuable insights by highlighting the apparent diffusion coefficient (ADC) of periprostatic fat as a potential functional imaging biomarker associated with adverse outcomes in prostate cancer (PCa). This innovative approach adds a physiological dimension to imaging interpretation, potentially enhancing traditional risk stratification methods.

While the study is both timely and thought-provoking, several limitations not addressed by the authors warrant further discussion to enhance the translational applicability of their findings. First, the analysis did not adjust for key systemic metabolic factors—such as body mass index (BMI), insulin resistance, lipid profile, and chronic inflammation—which are known to influence adipose tissue characteristics (2). These factors may independently affect ADC values, potentially confounding their relationship with tumor aggressiveness. Future studies should incorporate these variables to more accurately assess the independent prognostic value of periprostatic fat ADC.

Second, ADC values were derived from a single small region of interest (ROI) placed in the anterior periprostatic fat, assuming tissue homogeneity. However, adipose tissue can be spatially heterogeneous, particularly in patients with obesity or metabolic syndrome, where regional variations in fat composition and inflammation are common. Employing multi-slice or volumetric ADC analysis across anterior, lateral, and posterior regions could provide a more comprehensive and representative assessment of periprostatic fat, thereby enhancing the robustness and generalizability of this imaging biomarker (3, 4).

Third, while the proposed ADC-based approach is promising, its clinical implementation hinges on measurement reproducibility and operational simplicity. The current study does not evaluate inter-observer agreement or outline standardization procedures. In this context, recent advances in artificial intelligence (AI), radiomics, and machine learning offer promising avenues to improve measurement consistency and predictive accuracy (5, 6). Automated segmentation, texture analysis, and AI-driven risk models could minimize reader variability and facilitate the development of reproducible, clinically actionable tools for the early identification of aggressive PCa phenotypes.

In conclusion, Vieira et al. introduce a potentially impactful imaging biomarker for prostate cancer risk stratification. To maximize its clinical utility, future research should address metabolic confounding, adopt more extensive imaging protocols, and leverage AI-based technologies to improve reproducibility and integration into clinical workflows. These steps are essential for advancing personalized care in prostate cancer management.

The Authors
